# A revision of the genus *Antepione* Packard with description of the new genus *Pionenta* Ferris (Lepidoptera, Geometridae, Ennominae)

**DOI:** 10.3897/zookeys.71.789

**Published:** 2010-12-14

**Authors:** Clifford D. Ferris

**Affiliations:** 5405 Bill Nye Ave., R.R. 3, Laramie, WY 82070, USA, cdferris@uwyo.edu. Research Associate: McGuire Center for Lepidoptera and Biodiversity, Florida Museum of Natural History, University of Florida, Gainesville, FL; C. P. Gillette Museum of Arthropod Diversity, Colorado State University, Ft. Collins, CO; Florida State Collection of Arthropods, Gainesville, FL

**Keywords:** *Antepione*, Arizona, Colorado, Costa Rica, Ennominae, Geometridae, Guatemala, Lepidoptera, Mexico, New Mexico, nomenclature, North America, *Pionenta*, taxonomy, Texas

## Abstract

Based on genitalic studies, the new genus Pionenta is established for two taxa formerly placed under Antepione. The taxa *hewesata* and *ochreata* (and previously associated synonyms) are now synonomized as Pionenta ochreata. Three species of Antepione are now recognized: Antepione thisoaria, Antepione imitata, Antepione tiselaaria with the taxa *comstocki*, *constans*, and *indiscretata* synonomized under Antepione imitata. No new species are described. Adults and genitalia are illustrated, including type specimens.

## Introduction

A genitalic study of the eight species recognized by Parsons et al. (1999) associated with genus Antepione uncovered two distinct and quite different forms in both the male and female genitalia. This situation was alluded to by Pitkin (2002: 283), who excluded Antepione ochreata (Hulst) in her treatment of the genus. In Antepione, as restricted herein, the male genitalia lack a furca, and the female genitalia lack a signum. I recognize three species of Antepione. Antepione thisoaria (Guenée) is widely distributed in eastern North America with additional records for Mexico, Guatemala and Costa Rica. Antepione imitata occurs in the southwestern United States from Texas to Arizona. Antepione tiselaaria (Dyar) ranges from central Mexico to Costa Rica. In the genus Pionenta, as subsequently described, I recognize only one species, Pionenta ochreata. The male genitalia have a stubby robust furca, and the female genitalia a single large stellate signum. The species assigned to both genera are sexually dimorphic and extremely polyphenic, which, lacking genitalic examination, historically apparently led to the descriptions of multiple taxa. My field collections of multiple specimens in ultraviolet light traps at single sites and subsequent genitalic dissections allowed me to assess variation. The range of Pionenta is southwestern New Mexico and southeastern Arizona. It most likely occurs in contiguous northern Mexico, but I have found no records.

## Materials and Methods

### Repository abbreviations

AMNHAmerican Museum of Natural History, New York, NY, USA.

ANSPAcademy of Natural Sciences, Philadelphia, PA, USA.

BMNHThe Natural History Museum (formerly British Museum [Natural History]), London, UK.

CDFPersonal collection of Clifford D. Ferris, Laramie, WY, USA.

CMNHCarnegie Museum of Natural History, Pittsburgh, PA, USA.

CNCCanadian National Collection of Insects, Arachnids, and Nematodes, Ottawa, Ontario, Canada.

EMEEssig Museum of Entomology, University of California, Berkeley, CA, USA.

FMNHField Museum of Natural History, Chicago, IL, USA.

MCZMuseum of Comparative Zoology, Harvard University, Cambridge, MA, USA.

MNHNMuseum National d’Histoire Naturelle, Paris, France.

SEMSnow Entomological Museum Collection, University of Kansas, Lawrence, KS, USA.

USNM	National Museum of Natural History [formerly United States National Museum], Washington, District of Columbia, USA.

### Methods and general terminology.

Terms for genital structures and wing markings follow Ferris and Schmidt (2010).

### Description abbreviations

AMLAntemedial line.

DFWDorsal forewing.

DHWDorsal hindwing.

FWLForewing length, measured along costa from base to apex.

MBmedial band = area between DFW AML and PML.

PMLPostmedial line.

TLType locality.

### Key to genera

(based on DFW pattern and genitalia)

**Table:** 

1	DFW triangular costal dark patch present; male genitalia lack furca; female genitalia lack colliculum and signum	Antepione
–	DFW triangular costal dark patch absent; male genitalia with robust stubby furca; female genitalia with colliculum and signum	Pionenta ochreata

### Key to Antepione species

(based on genitalia)

**Table:** 

1	Male genitalia: apical region of valva lacks spines. Female genitalia: corpus bursa oblong and initially swollen with membranous anterior sac	Antepione thisoaria
–	Not as above	**2**
2	Male genitalia: valva rounded at apex with 3 long robust spines and additional fine setae. Female genitalia: corpus bursae long and cylindrical with membranous anterior sac	Antepione imitata
–	Not as above	**3**
3	Male genitalia: valva rounded at apex with multiple short slender translucent spines over most of surface excepting toward base. Female genitalia unknown to author	Antepione tiselaaria

### Antepione Packard, 1876: 459, 483

Epione depontanata Grote, 1864. Location of type unknown; originally placed in ANSP. Described from Maryland, USA.

### Mimogonodes  Warren, 1895

Mimogonodes constricta Warren, 1895 [BMNH].

#### Diagnosis.

**Adults.** Medium sized (FWL 13–21 mm) basically ochreous-colored moths with variable markings on DFW. DFW outer margin angulate at vein M_3_. Separation from similar genera is by the combination of characters: filiform antennae; male genitalia with stout tapered decurved uncus, valvae with even outer margins lacking projections, absence of furca; female genitalia without colliculum and signum.

#### Description.

**Adults.** Sexually dimorphic and sexes polyphenic; FWL 13–21 mm. Antenna simple, more slender in females. *Head* – Dark ochreous speckled with darker scales, concolorous collar; labial palpi broad, barely extending beyond frons, ochreous speckled with darker scales. *Thorax, abdomen, legs* – Ochreous or pale tan as in wings with widely scattered small brown scales. *Wings* – FW outer margin arcuate at vein M_3_ and HW; DFW apex acute to falcate. Usually obscure narrow dark DFW submarginal band; small dark discal spots both wings. **Males.** Dorsal color varies from gray, medium ochreous to medium brown. DFW AML and PML variable from pronounced and dark to broken and indistinct; medial band concolorous with remainder of wing, or paler and yellowish; a dark triangular patch with blunted or acute apex, with or without pale oblong spot, located along costa distad of PML. DHW with dark narrow medial band varying individually from dark to indistinct. Ventrally paler with dorsal maculation repeated, usually with less intensity. **Females**. Dorsal color varies from yellow through pale ochreous to medium ochreous and gray. Crosslines usually indistinct. DFW triangular patch as in males, PML above inner margin expanded into two large oblong brown spots. Ventrally paler with dorsal maculation repeated, usually with less intensity. *Male genitalia* – Uncus stout, slightly decurved, tapering to a rounded tip; medial gnathos with a few small teeth; valva rounded at apex; anellus with small spines; aedeagus truncate with one large oblong cornutus near base of vesica. *Female genitalia* – Apophyses long, slender; posterior apophyses ca. 1.8 × anterior apophyses; colliculum absent; ductus bursae ridged, short, partially sclerotized at posterior end; corpus bursae without signum, oblong with membranous anterior sac; ductus seminalis originates at top of ductus bursae.

#### Remarks.

McDunnough (1938) treated Epione depontanata and Heterolocha sulphurata Packard, 1876 as synonyms of Hyperythra arcasaria Walker, 1860. Forbes (1948: 108) placed Hyperythra arcasaria as a synonym of Heterolocha thisoaria (Guenée, 1858), which he then placed as Sabulodes thisoaria, and lumped several genera, including Antepione, under Sabulodes Guenée (1858). Subsequent authors (Ferguson 1983; Covell 1984; McGuffin 1987) restored Antepione as a separate genus. Ferguson recognized the species: *comstocki* Sperry; *hewesata* Sperry; *imitata*, H. Edwards.; *indiscretata*, (H. Edwards); *ochreata* (Hulst); *thisoaria* (Guenée); *tiselaaria* (Dyar). The geographic range of the genus includes eastern North America, the southwestern United States, portions of Mexico, Costa Rica and Guatemala.

## Systematics

### Antepione thisoaria

(Guenée, 1857 [1858])

Figs 1 11–19 59

Antepione sulphurata Packard 1876: 484
Epione depontanata Grote 1864: 90
Eutrapela furciferata Packard 1876: 559
Gonopteryx rhomboidaria Oberthür 1912: 246, pl. 148, f. 1401
Heterolocha sulphurata Packard 1873: 79
Heterolocha thisoaria Guenée 1857 [1858]: 106.
Hyperythra arcasaria Walker 1860: 131
Mimogonodes constricta Warren 1895: 149
Sabulodes thisoaria Forbes 1948: 108
Tetracis azonax Druce 1892: 54, pl. 46, f. 8
Tetracis rivulata Warren 1897: 506

#### Type material.

**Figures 1–4.**
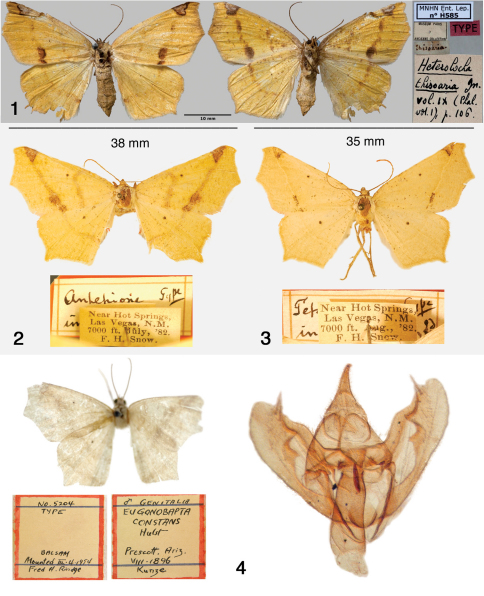
Antepione species. **1** Antepione thisoaria HT (dorsal and ventral) with pin labels (MNHN photo) **2** Antepione imitata HT with pin labels (SEMC photo) **3** Antepione (Tetracis) indiscretata HT with pin labels (SEMC photo) **4** Antepione (Eugonobapta) constans HT, adult, pin labels (AMNH photo) and male genitalia. The balsam embedding medium has fogged with age producing the apparent lack of focus in the genitalia photo.

Female HT (Fig. 1), country of origin not stated [MNHN].

#### Fixation of type locality.

The Central American taxa were not recognized and described until 1892 (*azonax*) and 1912 (*rhomboidaria*). On this basis, I infer that specimens from this region were not available to Guenée in 1857 when he described *thisoaria*, and that the holotype was collected in eastern North America. In habitus, the HT matches exactly female specimens of the *sulphuraria/sulphurata* form. The HT was most probably collected in the Middle Atlantic region. I hereby fix the type locality as eastern North America. Based on my research, it appears that Forbes (1948) was the first to use the name *thisoaria* in the North American fauna.

#### Other material examined.

84 specimens (a few by photograph) from Alabama, Indiana, Kentucky, Mexico, Michigan, New Jersey, New York, Nova Scotia, Pennsylvania, Quebec, Tennessee, Virginia. Additional distribution records were obtained from individuals and several museums, including 439 from the Carnegie Museum of Natural History, Pittsburgh, PA.

#### Diagnosis.

Antepione thisoaria is most easily separated from Antepione imitata based on geography. It does not occur west of the 95th parallel, while Antepione imitata extends eastward only to west Texas, and is not recorded from Central America. In the male genitalia, the apical region of the valva lacks spines, which are present in the valva of *imitata*. In the female genitalia, the corpus bursae is initially swollen while not so in Antepione imitata.

#### Description.

**Adults.** As described above for the genus. **Genitalia.** Figs 17–19. Two dissections (male and female) by author; illustrations in McGuffin (1987, Figs 242g, 245e); Pitkin (2002, Figs 202, 460).  Uncus stout, slightly decurved, tapering to a rounded tip; gnathos with unjoined slender arms, medial gnathos with a few small teeth; valva rounded at apex without spines*Male genitalia* –, produced ventral ridge forming two short projections; anellus with two sclerotized spinose lobes; aedeagus truncate with one large narrow elliptical cornutus near base of vesica. *Female genitalia* – Apophyses long, slender; posterior apophyses ca. 1.8 × anterior apophyses; ductus bursae ridged, moderately short, partially sclerotized at posterior; corpus bursae without signum, corpus bursae without signum, oblong and initially swollen with membranous anterior sac; ductus seminalis originates at top of ductus bursae.

#### Biology and distribution

(Fig. 59)**.** McGuffin (1987: 88–89) described the early stages and cited three specific larval hosts: Alnus rugosa (Du Roi) Spreng; Physocarpus opulifolius (L.) Maxim; Prunus serotina Ehrh. Various additional larval hosts are reported in the literature in the families Aceraceae, Anacardiaceae, Betulaceae, Ebenaceae, and Rosaceae. The last instar larva was illustrated by Wagner et al. (2001, p. 155) and Wagner (2005, p. 195). Adults fly April–May with an occasional mid-March and mid-June record, July–August with occasional September to mid-October records. There is one generation in Canada, and at least two southward. The distribution map (Fig. 59) represents the data that I was able to locate. The heavy distribution in Pennsylvania reflects intensive collecting in that state by CMNH personnel and volunteers. Undoubtedly similar efforts in neighboring areas should produce additional records. The overall range of this species is: in **CANADA** from Nova Scotia to Manitoba; in the **UNITED** **STATES** (county records in parentheses) then south and west to the Gulf states to the 95th parallel, including **Alabama** (Bibb, DeKalb, Jackson, Madison, Monroe), **Arkansas** (Logan, Montgomery, Polk, Scott, Washington), **Connecticut** (Fairfield, Hartford, New Haven, New London, Tolland, Windham), **Georgia** (Cherokee, Rabun), **Illinois** (Cook, Macon), **Indiana** (Elkhart, Jackson, Jasper, Lagrange, Laporte, Monroe,  Newton, Perry, Pulaski, St. Joseph), **Iowa** (Johnson, Monroe), **Kansas** (Crawford), **Kentucky** (Bell, Boone, Bracken, Bulitt, Calloway, Carter, Fayette, Graves, Harlan, Jefferson, Madison, McCracken, Meade, Menifee, Metcalfe, Morgan, Muhlenberg, Oldham, Owsley, Powell, Rowan, Russell, see Covell 1999), **Louisiana** (Feliciana Parish), **Maine** (Aroostook, Franklin, Oxford, Penobscot, Piscataquis), **Maryland** (Allegheny, Anne Arundel, Baltimore, Cecil, Garrett, Harford, Howard, Washington, Worcester), **Massachusetts** (Berkshire, Dukes, Essex, Middlesex, Nantucket), **Michigan** (Berrien, Cass, Otsego), **Minnesota** (Houston), **Mississippi** (Franklin, George, Grenada, Harrison, Kemper, Lee, Marshall, Oktibbeha, Pike, Pontotoc, Tishomingo, Union, Warren, Webster, Winston), **Missouri** (Barry, Benton, Camden, Cape Girardeau, Carter, Greene, Jasper, Lafayette, Lewis, Madison, Morgan, Newton, Stoddard, Warren, Wayne), **Nebraska** (Cass), **New Jersey** (Burlington, Essex, Gloucester, Morris, Passaic, Sussex, Union, Warren), **New Hampshire** (Rockingham), **New York** (Albany, Kings, Queens, Nassau, Suffolk, Westchester), **North Carolina** (Allegheny, Ashe, Avery, Stokes, Swain, Transylvania), **Ohio** (Adams, Ashland, Ross, Wayne), **Oklahoma** (Cherokee, see Nelson 2010), **Pennsylvania** (Adams, Allegheny, Armstrong, Beaver, Bedford, Berks, Blair, Bucks, Butler, Centre, Chester, Clearfield, Crawford, Dauphin, Fulton, Greene, Huntingdon, Lawrence, Northumberland, Perry, Somerset, Washington, Westmoreland, York), **Rhode Island** (Washington), **South Carolin**a (Greenville), **Tennessee** (Louden, Wilson), **Virginia** (Augusta, Carroll, Giles), **West Virginia** (Cabell, Grant, Greenbrier, Hampshire, Mason, Monongalia, Monroe, Pendleton, Randolph, Roane, Wyoming); **MEXICO** (Michoacan state); **CENTRAL AMERICA** in Costa Rica and Guatemala (Pitkin et al. 1996). Covell (1984) stated the westward range of the species to Texas. I have been unable to confirm Texas from museum records. Forbes (1948) stated: “. . . varieties in Colorado, Texas, and Arizona.” His “varieties” are assumed to be Antepione imitata. Although the distribution map (Fig. 59) suggests occurrence of *thisoaria* in Florida, Vermont and Wisconsin, no records were found.

#### Remarks.

The gray spring form of the moth (Figs 11, 15) was described by Packard as the species *furciferata*. The male (Fig. 14) represents the summer form *arcasaria*, and the female (Fig. 16) represents the summer form *sulphuraria* = *sulphurata*. Packard (1876) redescribed Heterolocha sulphuraria Packard, 1873 as Antepione sulphurata. Once barcoding data are available, the disjunct distributions of Mexican and Central American populations may ultimately prove to be separate species, in which case the name *azonax* Druce, 1892 (Costa Rica, Guatemala) is available and has date priority over *rhomboidaria* Oberthür, 1912 (Costa Rica) and *rivulata* Warren, 1897 (Costa Rica). The two female specimens in the CNC from Tuxpan, Michoacan, Mexico are exact matches for the *sulphuraria*/*sulphurata* phenotype and were collected in early August, 1959.

### Antepione imitata

Edwards, 1884

Figs 2 5 20–33 59

Antepione comstocki Sperry 1939, syn. n.
Antepione costinotata Taylor 1905
Eugonobapta constans Hulst 1898, syn. n.
Metanema vanusaria Strecker 1899: 6, syn. rev.
Tetracis indiscretata Edwards 1884: 48, syn. n.

#### Type material.

Female HT (Fig. 2), New Mexico, [San Miguel Co.], Las Vegas, July, 1882. [SEMC].

**Figures 5–10.**
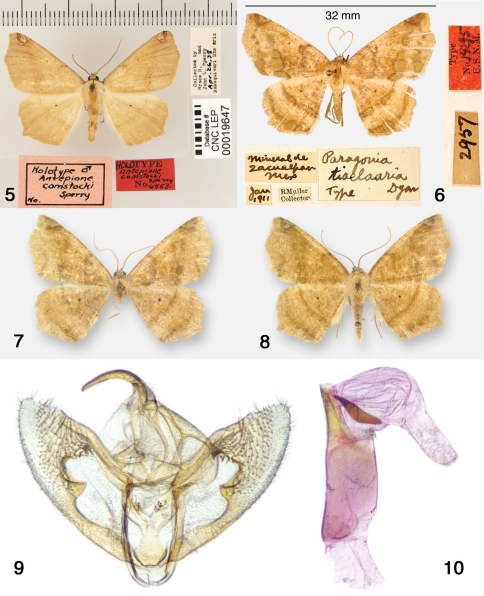
Antepione species. **5** Antepione comstocki HT with pin labels (CNC photo) **6–10** Antepione tiselaaria. **6** HT with pin labels (USNM photo) **7–8** adult males **9** male genitalic capsule, aedeagus removed **10** aedeagus with vesica everted.

Antepione comstocki male HT (Fig. 5), Arizona, [Pima Co.], Baboquivari Mts., 26 April, 1938 [CNC]. Tetracis indiscretata female HT (Fig. 3), New Mexico, [San Miguel Co.], Las Vegas, August, 1882 [SEMC]. Eugonobapta constans male HT (Fig. 4), Arizona, [Yavapai Co.], Prescott, August, 1896 [AMNH].

#### Other material examined.

145 specimens in [CDF] from Arizona, Colorado and New Mexico; additional material (some by photographs) from Arizona (including a reared series), Colorado, New Mexico, Texas, Mexico.

#### Diagnosis.

Antepione imitata is most easily separated from Antepione thisoaria based on geography. It does not occur east of west Texas and is not recorded from Central America, while Antepione thisoaria extends west only to the 95th parallel. In the male genitalia, the apical region of the valva exhibits 3 long robust spines and additional fine setae, which are not present in the valva of *thisoaria*. In the female genitalia, the corpus bursae is not initially swollen as in Antepione thisoaria.

#### Description.

**Adults.** As described above for the genus. **Genitalia.** Figs 4, 31–33. Dissections 8m, 2f comprising full range of phenotypes). *Male genitalia* – Uncus stout, slightly decurved, tapering to a rounded tip; gnathos with unjoined slender arms, medial gnathos with a few small teeth; valva rounded at apex with 3 long robust spines and additional fine setae, produced ventral ridge forming two short projections; anellus with two sclerotized spinose lobes; aedeagus truncate with one large oblong cornutus near base of vesica. *Female genitalia* – Apophyses long, slender; posterior apophyses ca. 1.8 × anterior apophyses; ductus bursae ridged, short, partially sclerotized at posterior; corpus bursae without signum, long and cylindrical with membranous anterior sac; ductus seminalis originates at top of ductus bursae.

**Figures 11–19.**
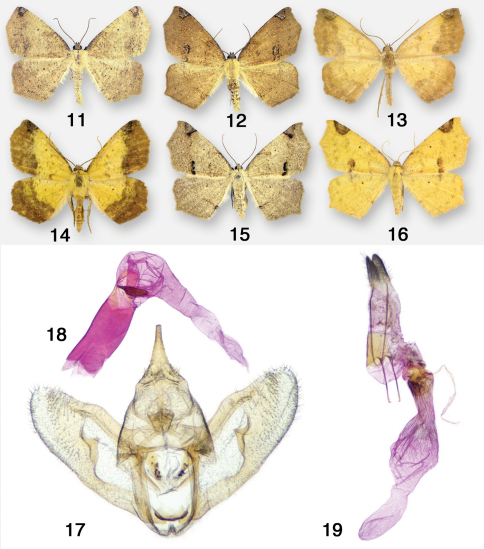
Antepione thisoaria. **11–14** adult males **15–16** adult females **17** male genitalic capsule, aedeagus removed **18** aedeagus with vesica everted **19** female genitalia.

**Figures 20–30.**
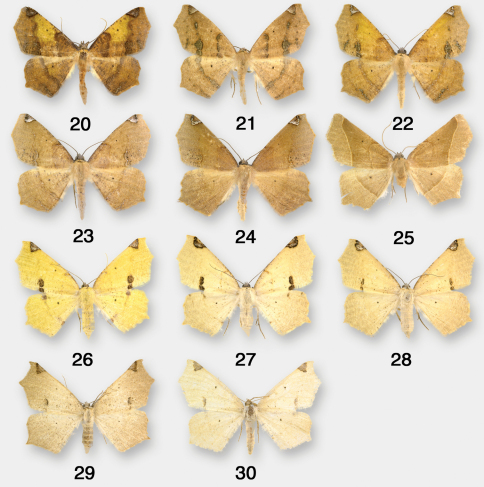
Antepione imitata adults. **20–25** males **26–30** females.

**Figures 31–33.**
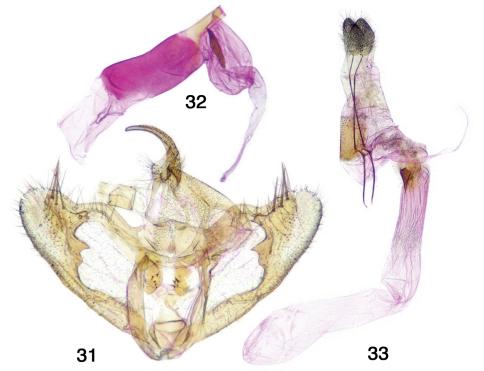
Antepione imitata genitalia. **31** male genitalic capsule, aedeagus removed **32** aedeagus with vesica everted **33** female genitalia.

**Remarks.** One male specimen (Fig. 25) of the *comstocki* phenotype examined from Las Animas Co., Colorado lacks the characteristic DFW costal triangular patch, causing it to resemble superficially the *ligata* form of Pionenta ochreata. The male genitalia, however, are typical of Antepione imitata.

#### Biology and distribution

(Fig. 59)**.** Noel McFarland (Hereford, AZ) reared the species on Ribes aureum Push. from ova from an adult female of the nearly uniformly brownish-ochreous April–May generation; adults emerged June–July. The resulting adults are of the form with yellow females and males in which the DFW medial band has a yellow flush. Based on my field studies over many years in southeastern Arizona and southwestern New Mexico and McFarland’s reared material, there appear to be three generations in southeastern Arizona and Southwestern New Mexico. There is a strong early flight starting in April and early May, with a weaker flight in late June into July, and another strong flight beginning in mid-August after the monsoonal rains with a few individuals into early October. This species ranges from west **Texas** (Brewster, Culberson, Jeff Davis), **Colorado** (Delta, La Plata, Las Animas), **New Mexico** (Grant, Harding, Hidalgo, San Miguel), to southern **Arizona** (Cochise, Gila, Pima, Santa Cruz). A typical male specimen was examined [CMNH] with the collection data: **Mexico:** Coahuila, Sierra La Madera, upper Canada Desiderio, 15–17 March 1985, 27–08N, 102–31W, 1810m, J. Rawlins, S. Thompson. This locality is essentially due south of the western Texas records, and one might anticipate that with further collecting Antepione imitata will prove to be widespread in northern Mexico. It is generally associated with riparian canyons up to 6000’ (1830m).

#### Discussion.

As is also the case with Antepione thisoaria, most spring individuals of Antepione imitata are rather drab in appearance with lightly maculated brownish males (the *comstocki* phenotype) and pale creamy colored or ochreous females. The strongly maculated males and yellow females appear in the later generations in company with the rather drab early-season phenotypes. In his original descriptions of *imitata* and *indiscretata*, Edwards provided no insight as to why he assigned *imitata* to Antepione and *indiscretata* to Tetracis. Both taxa are described on the same page with the description of *imitata* preceding that of *indiscretata*. He characterized the color of *imitata* as similar to the yellow *sulphurata* phenotype of *thisoaria*, and *indiscretata* as “Ochraceus drab.” Over the years the type specimens have faded to some extent so that they now appear nearly identical in color, the only difference being the extent of the dark maculation. The name *constans* appears to have been applied to the heavily maculated male phenotype, as best can be determined from the poor condition of the HT.

### Antepione tiselaaria

(Dyar, 1912)

Figs 6–10

Paragonia tiselaaria Dyar 1912

#### Type material.

Male HT (Fig. 6), Mexico, Minerale de Zacualpan, January, 1911 [USNM]. Comment: Dyar (1912: 87) stated the type locality only as “Zacualpan” and not “Minerale de Zacualpan” as shown on the specimen label. I interpret the label to mean the Zacualpan mining region located in the state of Morelos south of Mexico City, today still an active silver mining district.

#### Other material examined

(Figs 7–10). MEXICO. Puebla, 2 mi. SW Tehuacan, 5300’, 4.x.1975, Powell (1m, dissected); same, 5.x.1975, J. Powell (1m) [EME].

#### Diagnosis.

Females not known to the author. Mexican specimens of Antepione tiselaaria males are most easily separated from Antepione imitata based on geography, since the latter species does not penetrate south to central Mexico. In Costa Rica, where Antepione thisoaria is also reported, Antepione tiselaaria manifests a more orange-brown overall color than the drab ochreous-gray form of *thisoaria*. In the male genitalia, the apical region of the valva is covered with multiple short slender translucent spines over most of the surface except toward the base; spines are absent in the valva of Antepione thisoaria, and 3 long robust spines occur in Antepione imitata.

#### Description.

**Adults.** Only males were available for examination. As described above for the genus, other than the wings. FWL 17–18 mm. *Wings* – FW outer margin arcuate (roundly produced about) vein M_3_ and HW; DFW apex sightly acute, not falcate. Dorsal color pale orange-brown-ochreous with darker maculation. AML a narrow band centrally with a few paler scales, PML an interrupted band with irregular edges and centrally paler, widening substantially approaching inner margin; MB not clearly defined with splotchy brown maculation over paler ground color; a dark triangular patch with blunted or acute apex, with or without pale oblong spot, located along costa distad of PML; small dark discal spots FW and HW. Ventrally paler with dorsal maculation repeated with slightly less intensity. **Male genitalia**. Figs 9–10. Dissection 1m. Uncus stout, slightly decurved, tapering to a rounded tip; gnathos with unjoined slender arms, medial gnathos with a few very small dark teeth; valva rounded at apex with multiple short slender translucent spines over most of the surface excepting toward the base, produced ventral ridge forming one large and one short projection; anellus with two sclerotized spinose lobes; aedeagus truncate with one large (equal to diameter of aedeagus shaft) oblong triangular cornutus near base of vesica; fully everted vesica initially spherical becoming a tapered tube.

#### Biology and distribution.

Early stages unknown. Current distribution records are for the Mexican states of Morelos and Puebla, and Costa Rica.

### Pionenta

Ferris gen. n.

urn:lsid:zoobank.org:act:95BF80CA-F55F-40D9-B877-A25252635770

#### Type species:

Antepione ochreata Hulst, 1898.

#### Etymology.

Pionenta is a masculine anagram of Antepione.

#### Diagnosis.

Pionenta ochreata lacks the DFW triangular costal patch found in all species of Antepione. The well-developed DFW AML and PML form a wedge-shaped medial band absent in Antepione. The robust centered furca in the male genitalia and large stellate signum in the female genitalia of Pionenta are absent in Antepione.

#### Description.

**Adults.** Sexually dimorphic and both sexes are polyphenic; FWL 14–19 mm. Antenna simple. *Head* – Uniformly ochreous, collar concolorous; labial palpi relatively narrow, slightly upcurved, ochreous, barely extending beyond frons. *Thorax, abdomen, legs* – Uniformly colored as in ground color of wings with a few widely scattered small brown scales on legs. *Wings –* Outer margin arcuate FW (about M_3_) and HW; DFW apex normally sightly falcate. Wing color variable from pale creamy white to ochreous tan. AML and PML narrow and brown (occasionally reddish-brown), PML continues on DHW as medial line; AML with narrow pale shading basad, PML with narrow pale shading distad. MB trapezoidal tapering inward from costa to inner margin. Small dark discal spots present FW and HW. Scattered dark patches may be present basally and submarginally on DFW, and submarginally on DWH. Ventrally paler with dorsal maculation only weakly repeated. *Male genitalia* (7 dissections by author, additional museum slides examined) – Uncus stout, slightly decurved, tapering at apex to a rounded tip; gnathos v-shaped with well-sclerotized edges, medially a sharp upcurved tip with numerous very small teeth; valva rounded at apex, but with blunt triangular projection at end of sclerotized costa; anellus membranous without spines or setae, with central robust cylindrical furca covered by numerous short spines on rounded apex; aedeagus truncate with two long sclerotized pointed extensions from apical margin and a variable patch of apparently deciduous dark setae near base of otherwise membranous short cylindrical vesica. *Female genitalia* (6 dissections) – Posterior apophyses short, anterior apophyses much reduced ca. 0.4 × posterior apophyses; sterigma well sclerotized; posterior margin of lamella antevaginalis rounded at ends with central depression; well-defined colliculum; partially ridged short ductus bursae opens into ovoid membranous corpus bursae; one large centrally located oval stellate signum; ductus seminalis originates immediately below colliculum.

### Pionenta ochreata

Hulst, 1898

Figs 34–58 60

Antepione hewesata Sperry 1948, syn. n.
Sabulodes arizonata Taylor 1905, syn. rev.
Sabulodes dyari Grossbeck 1908, syn. rev.
Sabulodes ligata Grossbeck 1908, syn. rev.

#### Type material.

**Figures 34–39.**
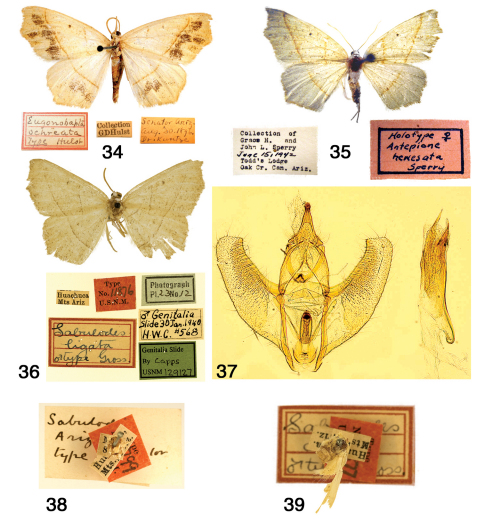
Pionenta ochreata. **34** Pionenta ochreata HT with pin labels (AMNH photo) **35** Pionenta (Antepione) hewesata HT with pin labels (AMNH photo) **36–37** Pionenta (Sabulodes) ligata **36** HT with pin labels **37** genitalia **38** Pionenta (Sabulodes) arizonata HT with pin labels **39** Pionenta (Sabulodes) dyari HT with pin labels. (36–39 USNM photos).

Male HT (Fig. 34), Arizona, Senator [probably Senator Mine, Yavapai Co.] [AMNH].

Antepione hewesata female HT (Fig. 35), Arizona [Coconino Co.], Oak Creek Canyon, Todd’s Lodge, [AMNH]. Sabulodes arizonata male HT (Fig. 38), Arizona [Cochise Co.], Huachuca Mts. [USNM]. Sabulodes dyari male HT (Fig. 39), Arizona [Cochise Co.], Huachuca Mts. [USNM]. Sabulodes ligata male HT (Figs 36–37), Arizona [Cochise Co.], Huachuca Mts. [USNM].

#### Other material examined.

135 specimens in [CDF] from Arizona and New Mexico; 61 additional specimens (some by photographs) from Arizona.

#### Diagnosis.

As for genus.

#### Description.

General description as for genus.

**Figures 40–53.**
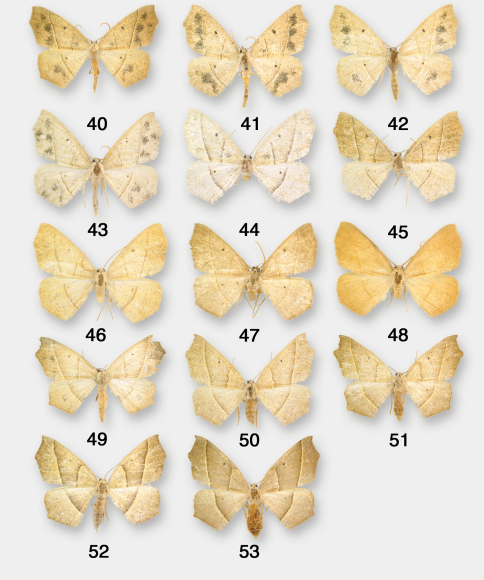
Pionenta ochreata adults. **40–48** males **49–53** females.

**Figures 54–58.**
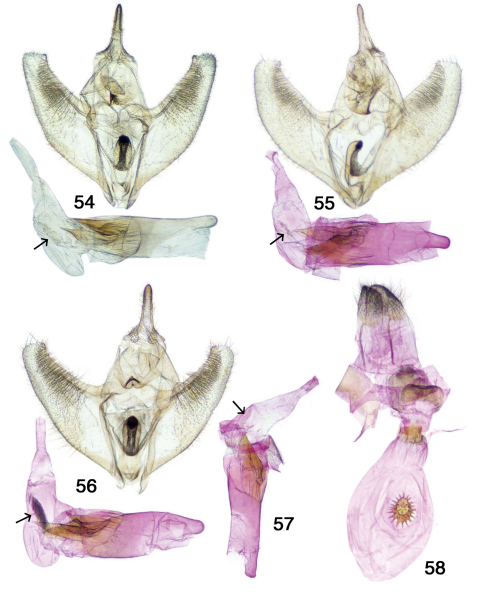
Pionenta ochreata genitalia. **54–57** male genitalic capsules, adeagii removed and adeagii with vesicas everted (arrows point to deciduous setae) **58** female genitalia.

#### Remarks.

Antepione ochreata has two distinct phenotypes. The male form with pale ochreous wing color and varying numbers of multiple dark patches (Figs 34, 40–43) was described as *ochreata*. The female described as *hewesata* (Figs 35, 44) is intermediate between typical *ochreata* and the brownish-tan phenotype without dark patches described as *ligata* (Figs 36, 46, 50–53) which is the usual female form based on my field experience and examination of museum material. Regarding the taxa *arizonata* and *dyari*, apparently some years ago an accident occurred with a drawer containing type specimens and they were badly damaged. Figs 38 and 39 illustrate what remains of these two specimens. Their associated genitalia slides were not damaged and the preparations agree with Figs 37, 54–57.

#### Biology and distribution

(Fig. 60). Early stages unknown. Adults from mid-May to August in riparian canyons and dry coniferous forest to 8400’ (2560m); probably more than one generation. Collection records include **Arizona** (Cochise, Coconino, Pima, Santa. Cruz, Yavapai), **New Mexico** (Grant).

**Figure 59.**
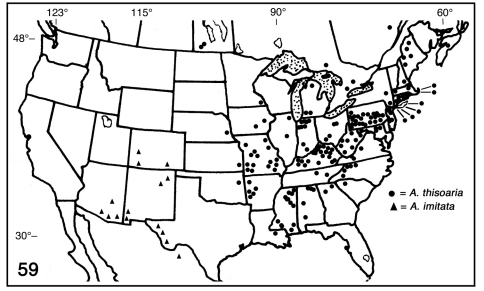
Partial distribution map for Antepione thisoaria and Antepione imitata.

**Figure 60.**
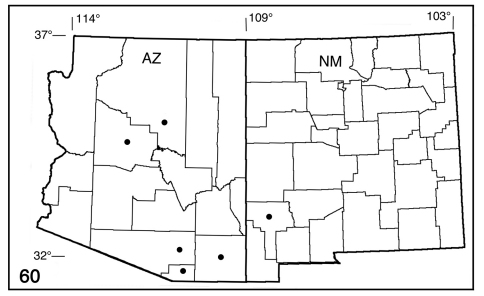
Distribution map for Pionenta ochreata (counties only).

#### Discussion.

Based on the male and female genitalia, Pionenta is closely related to Tetracis. The male genitalia of both genera possess a well defined central furca. The female genitalia of both genera possess a well defined colliculum and prominent single signum. The gnathos in Pionenta does not have a quadrate dorso-caudal margin with two to four (occasionally five) widely separated, dorsally projecting teeth as found in Tetracis (Ferris & Schmidt, 2010). Once barcoding of the North America geometrid genera has been completed, the relationship of Pionenta can be established.

## Supplementary Material

XML Treatment for Antepione thisoaria

XML Treatment for Antepione imitata

XML Treatment for Antepione tiselaaria

XML Treatment for Pionenta

XML Treatment for Pionenta ochreata

## Appendix: Annotated checklist of the taxa assigned to Antepione and Pionenta

Antepione Packard, 1876

Antepione imitata H. Edwards, 1884, New Mexico, Las Vegas [HT female, SEMC]

Antepione comstocki Sperry, 1939, **syn. n.**, Arizona, Baboquivari Mts. [HT male, CNC]

Antepione constans (Hulst, 1898), **syn. n.**, Arizona, Prescott [HT male, AMNH]

Antepione costinotata Taylor, 1906, Colorado, Durango [HT female, USNM] Note 1

Antepione indiscretata (H. Edwards), 1884, **syn. n.**, New Mexico, Las Vegas [HT female, SEMC]

Antepione vanusaria (Strecker, 1899), **syn. rev.**, New Mexico [HT male, FMNH]

Antepione thisoaria Guenée, 1857 [1858], fixed herein as eastern North America [HT female, MNHN]

Antepione arcasaria (Walker, 1860), [HT female, BMNH]

Antepione azonax (Druce, 1892) Guatemala, San Geronimo; Costa Rica, Volcan de Irazu [ST female, BMNH] Note 2

Antepione constricta (Warren, 1895), ? South America [HT male, BMNH] Note 2

Antepione depontanata (Grote, 1864), Maryland [HT male] Note 3

Antepione fuciferata (Packard, 1876), New York [HT male, MCZ]

Antepione rhomboidaria (Oberthür, 1912), Costa Rica, San Jose [STs, BMNH] Note 2

Antepione rivulata (Warren, 1897), Costa Rica [HT female, BMNH] Note 2

Antepione sulphuraria (Packard, 1873), New York, West Farms; Middle States [HT female, MCZ]

Antepione sulphurata (Packard, 1876) Note 4

Antepione tiselaaria (Dyar. 1912), Mexico, Minerale de Zacualpan [HT male, USNM]

Pionenta Ferris, 2010, **gen. n.**

Pionenta ochreata (Hulst, 1898), **comb. n.**, Arizona, Senator [HT male, AMNH]

Pionenta arizonata (Taylor, 1905), **syn. rev.**, Arizona, Cochise Co., Huachuca Mts. [HT male, USNM]

Pionenta dyari (Grossbeck, 1908), **syn. rev.**, Arizona, Huachuca Mts. [HT male, USNM]

Pionenta hewesata (Sperry, 1948), **syn. n.**, Arizona, Oak Creek Canyon, Todd’s Lodge [HT female, AMNH]

Pionenta ligata (Grossbeck, 1908), **syn. rev.**, Arizona, Huachuca Mts. [HT male, USNM]

### Notes:

1.	Taylor stated in his original description that the HT was type number 9800 in the USNM. A recent attempt to locate the type failed, and it is presumed misplaced or lost. The TL was incorrectly stated as Prescott, Arizona in Parsons et al. (1999).

2.	Barcoding of these taxa may ultimately indicate species distinct from *thisoaria*.

3.	The type was originally placed in the collection of the Entomological Society of Philadelphia and subsequently in ANSP. In the 1960s, the bulk of the ANSP Lepidoptera collection went to CMNH. The type cannot be located in either ANSP or CMNH and is presumed lost.

4.	Packard (1876) redescribed Heterolocha sulphuraria Packard, 1873 as Antepione sulphurata.

## References

[B1] Covell CV Jr (1984) A field guide to the moths of eastern North America. Houghton Mifflin Co., Boston, MA, 496 pp.

[B2] Covell CV Jr (1999) The butterflies and moths (Lepidoptera) of Kentucky: an annotated checklist. Kentucky State Nature Preserves Commission Scientific and Technical Series 6, 220 pp.

[B3] Druce H (1892–1900) Biologia Centrali-Americana. Lepidoptera – Heterocera. 2, 622 pp.

[B4] Dyar HG (1912) Descriptions of new species and genera of Lepidoptera, chiefly from Mexico. Proceedings of the United States National Museum 42:39-106

[B5] Ferguson DC (1983)Geometridae. In: Hodges RW Dominick T Davis DR Ferguson DC Franclemont JG Munroe EG Powell JA (Eds) Check List of the Lepidoptera of America North of Mexico, 88–107

[B6] Forbes WTM (1948) Lepidoptera of New York and neighboring states. 2. Memoirs of the Cornell Agricultural Experiment Station 274, 263 pp.

[B7] Edwards H (1884) Descriptions of new species of N. Am. Heterocera Papilio 4: 11–19, 48

[B8] Ferris CD Schmidt BC (2010) Revision of the North American Genera *Tetracis* Guenée and Synonymization of *Synaxis* Hulst with Descriptions of Three New Species (Lepidoptera: Geometridae: Ennominae). Zootaxa 2347:1-36

[B9] Grote AR (1864) Descriptions of North American Lepidoptera. No. 3. Proceedings of the Entomological Society of Philadelphia 3:73-92

[B10] Grossbeck JA (1908) Additions to the list of North American Geometridae with notes on some described species. Journal of the Entomological Society of Washington 10:85-91

[B11] Guenée A (1857 [1858]) Vol. 9, Uranides et Phalénites 1. In: Boisduval JBAD de Guenée A (Eds) Histoire Naturelle des Insectes. Species Général des Lépidoptères, Roret, Paris, 551 pp.

[B12] Hodges RW Dominick T Davis DR Ferguson DC Franclemont JG Munroe EG Powell JA (1983) Check List of the Lepidoptera of America North of Mexico. E. W. Classey Ltd, London and The Wedge Entomological Research Foundation, Washington, xxiv + 284 pp.

[B13] Hulst GD (1898) Descriptions of new genera and species of the geometrina of North America. Transactions of the American Entomological Society 30:214-219

[B14] McDunnough J (1938) Check list of the Lepidoptera of Canada and the United States of America. Part 1, Macrolepidoptera. Memoirs of the Southern California Academy of Sciences, vol. 1, 272 pp.

[B15] McGuffin WC (1987) Guide to the Geometridae of Canada (Lepidoptera) II. Subfamily Ennominae 4. Memoirs of the Entomological Society of Canada, No. 138: 1–182

[B16] Nelson JM (2010) Oklahoma Moth Species by County. http://www.biosurvey,ou.edu/ok_butterfly.html

[B17] Oberthür C (1912) Révision des Phalénites décrites par Guenée dans le Species général des Lépidoptères (Tome IX) – Famille II. Ennomidae, Guenée. Études de Lépidoptérologie comparée 6: 223–307, 346–355, pls. 144–160.

[B18] Packard AS (1873) Descriptions of new American Phalaenidae. 5th Annual Report of the Trustees of the Peabody Academy of Science, 52–81

[B19] Packard AS (1876)A monograph of the geometrid moths or Phalaenidae of the United States. Report of the United States Geological Survey of the Territories 10, 607 pp, iv, 13 plates.

[B20] Parsons MS Scoble MJ Honey MR Pitkin LM Pitkin BR (1999) The Catalogue. In: MJ Scoble (Ed) Geometrid Moths of the World: a Catalogue (Lepidoptera, Geometridae). CSIRO Publishing, Collingwood. 2 vol. 1,016 pp. + 129 pp. + 12 9 pp.

[B21] Pitkin LM (2002) Neotropical ennomine moths: a review of the genera (Lepidoptera: Geometridae). Zoological Journal of the Linnean Society 135:121-401

[B22] Pitkin, LM Mora RA Scoble MJ (1996) A checklist to the Ennominae (Geometridae) of Costa Rica: taxonomy for a national biodiversity inventory. Gayana Zoologia 60:121-155

[B23] Sperry JL (1939) Two apparently new geometrids from the Southwest. Canadian Entomologist 71:262-263

[B24] Sperry JL (1948) Southwestern geometrid notes and new species II. Bulletin of the Brooklyn Entomological Society 43:88-93

[B25] Strecker H (1899) Lepidoptera, Rhopaloceres and Heteroceres, indigenous and exotic. Lep., Rhopal. and Het., Suppl. 2: 1 1 pp.

[B26] Taylor GW (1905) Some new Geometridae from Arizona. Journal of the New York Entomological Society 13:130-131

[B27] Wagner DL (2005) Caterpillars of eastern North America. Princeton University Press, Princeton, NJ, 512 pp.

[B28] Wagner DL Ferguson DC McCabe TL Reardon, RC (2001) Geometrid caterpillars of northeastern and Appalachian forests. U.S. Department of Agriculture, Forest Service, publication FHTET-2001–10, 237 pp.

[B29] Walker F (1860) List of the Specimens of Lepidopterous Insects in the Collection of the British Museum 20, London, 276 pp.

[B30] Warren W (1894) New genera and species of Geometridae. Novitates Zoologicae 1:366-466

[B31] Warren W (1895) New genera and species of Geometridae. Novitates Zoologicae 2:82-159

[B32] Warren W (1897) New genera and species of Geometridae. Novitates Zoologicae 4:408-507

